# Virulence properties of *Campylobacter jejuni* are enhanced by displaying a mycobacterial TlyA methylation pattern in its rRNA

**DOI:** 10.1111/cmi.13199

**Published:** 2020-03-24

**Authors:** Agnieszka Sałamaszyńska‐Guz, Izabela Serafińska, Paweł Bącal, Stephen Douthwaite

**Affiliations:** ^1^ Division of Microbiology, Department of Pre‐Clinical Sciences, Institute of Veterinary Medicine Warsaw University of Live Sciences – SGGW Warsaw Poland; ^2^ Laboratory of Theory and Applications of Electrodes, Faculty of Chemistry University of Warsaw Warsaw Poland; ^3^ Nałęcz Institute of Biocybernetics and Biomedical Engineering Polish Academy of Sciences Warsaw Poland; ^4^ Department of Biochemistry and Molecular Biology University of Southern Denmark Odense M Denmark

**Keywords:** bacterial motility, biofilms, capreomycin resistance, epithelial cell invasion, rRNA 2′‐*O*‐methylation

## Abstract

*Campylobacter jejuni* is a bacterial pathogen that is generally acquired as a zoonotic infection from poultry and animals. Adhesion of *C. jejuni* to human colorectal epithelial cells is weakened after loss of its *cj0588* gene. The Cj0588 protein belongs to the type I group of TlyA (TlyA^I^) enzymes, which 2′‐*O*‐methylate nucleotide C1920 in 23S rRNA. Slightly longer TlyA^II^ versions of the methyltransferase are found in actinobacterial species including *Mycobacterium tuberculosis*, and methylate not only C1920 but also nucleotide C1409 in 16S rRNA. Loss of TlyA function attenuates virulence of both *M. tuberculosis* and *C. jejuni*. We show here that the traits impaired in *C. jejuni* null strains can be rescued by complementation not only with the original *cj0588 (tlyA* ^*I*^ *)* but also with a mycobacterial *tlyA* ^*II*^ gene. There are, however, significant differences in the recombinant phenotypes. While *cj0588* restores motility, biofilm formation, adhesion to and invasion of human epithelial cells and stimulation of IL‐8 production in a *C. jejuni* null strain, several of these properties are further enhanced by the mycobacterial *tlyA* ^*II*^ gene, in some cases to twice the original wild‐type level. These findings strongly suggest that subtle changes in rRNA modification patterns can affect protein synthesis in a manner that has serious consequences for bacterial pathogenicity.

## 1 INTRODUCTION

Orthologs of TlyA proteins are expressed in a diverse range of bacterial pathogens including *Campylobacter jejuni* and *Mycobacterium* spp. and have been linked to various roles in pathogenesis including bacterial colonisation (Sałamaszyńska‐Guz et al., 2008; Hyatt, ter Huurne, van der Zeijst, & Joens, 1994; Martino et al., 2001; Zhang, Dorrell, Wren, & Farthingt, 2002), influence on the immune response of the host (Rahman et al., 2015), haemolysis (Monshupanee, 2013; Wren et al., 1998) and antibiotic resistance (Maus, Plikaytis, & Shinnick, 2005).

In actinobacterial species, which include *Mycobacterium tuberculosis*, TlyA enzymes belong to the type II group (TlyA^II^) and have the function of 2′‐*O*‐methylating nucleotide C1409 in 16S rRNA and nucleotide C1920 in 23S rRNA (Johansen, Maus, Plikaytis, & Douthwaite, 2006). Slightly shorter versions of TlyA truncated at their N‐ and C‐termini are found in *C. jejuni*, *Helicobacter pylori* and *Brachyspira hyodysenteriae*, and belong to the type I group of enzymes (TlyA^I^) that methylate only at 23S rRNA nucleotide C1920 (Monshupanee, Johansen, Dahlberg, & Douthwaite, 2012; Sałamaszyńska‐Guz et al., 2018).

Loss of TlyA in *C. jejuni* cells results in a wide range of defects including decreased ribosome subunit association, impeded motility and reduced biofilm formation, which collectively reduce virulence (Sałamaszyńska‐Guz et al., 2018). In addition, sensitivity to the antibiotic capreomycin is altered. Complementation with natively folded variants of TlyA containing point mutations that abolish methyltransferase activity showed that all the physiological defects were caused by loss of rRNA methylation rather than absence of the protein itself (Sałamaszyńska‐Guz et al., 2018). These findings indicate that TlyA influences the physiology and pathogenicity of *C. jejuni* solely through its rRNA methylation activity.

Studies on the mycobacterial TlyA^II^ and mutant derivatives of this enzyme showed that both the C1409 and C1920 methylations contribute to capreomycin binding (Monshupanee et al., 2012). These nucleotides are respectively located on the interface of the small and large ribosomal subunits (Yusupov et al., 2001) at the extremities of the binding site for capreomycin and the related tuberactinomycin drug, viomycin (Stanley, Blaha, Grodzicki, Strickler, & Steitz, 2010). While the connection between TlyA‐directed methylation and capreomycin/viomycin binding is immediately evident (Johansen et al., 2006), it remains less clear how the presence or absence of rRNA methylation would affect protein synthesis in a manner that alters pathogenic traits. Here, we address this question by equipping a *tlyA*‐null strain of *C. jejuni* with either an authentic copy its own *tlyA* ^*I*^ gene (*cj0588*) or the mycobacterial *tlyA* ^*II*^ gene. The influence of the different methylation patterns in the *C. jejuni* recombinants are shown to be linked to a range of parameters including cell motility, biofilm formation, adhesion to human epithelial cells, cell invasion and the ability to elicit an innate immune response in the host cell.

## 2 MATERIALS AND METHODS

### 2.1 Bacterial strains

The *C. jejuni* strains used in this study (Table 1) were grown under microaerobic conditions (BD GasPak EZ CO2 sachets, Becton Dickinson) at 37°C on brain–heart infusion (BHI) agar containing 5% (v/v) sheep blood, and in some cases supplemented with chloramphenicol at 20 μg/ml and/or kanamycin at 30 μg/ml.

**Table 1:** *C. jejuni* strains used in this study

Strains	Relevant characteristics	Source/reference
*C. jejuni* 81–176	Wild type strain (WT)	Korlath, Osterholm, Judy, Forfang, & Robinson, 1985
*C. jejuni* 81–176 Δ*cj0588*	Cm^r^, *cj0588* (*tlyA* ^*I*^) deletion mutant	Sałamaszyńska‐Guz et al., 2018
*C. jejuni* 81–176 Δ*cj0588*::*cj0588*	Cm^r^, Km^r^, *cj0588* deleted, complemented with *C. jejuni cj0588*	Sałamaszyńska‐Guz et al., 2018
*C. jejuni* 81–176 Δ*cj0588*::*cj0588*K188A	Cm^r^, Km^r^ *cj0588* deleted, complemented with K188A mutant *cj0588*	Sałamaszyńska‐Guz et al., 2018
*C. jejuni* 81–176 Δ*cj0588*::*Myco_tlyA* ^*II*^	Cm^r^, Km^r^, *cj0588* deleted, complemented with *Mycobacterim smegmatis* wild‐type *tlyA* ^*II*^	This study

### 2.2 Complementation of *C. jejuni cj0588*‐null strain

The *C. jejuni* 81–176 null mutant was complemented by inserting the *Mycobacterium smegmatis* wild‐type *tlyA* ^*II*^ into the 121‐bp intergenic region between *cj0652* and *cj0653c* (Javed et al., 2012; Wösten, Boeve, Koot, van Nuenen, & van der Zeijst, 1998) under control of the *C. jejuni cj0183* gene promoter (Sałamaszyńska‐Guz, Grodzik, & Klimuszko, 2013). This created strain 81‐176Δ*cj0588*::*Myco_tlyA* ^*II*^ (Table 1). All strain constructions were verified by polymerase chain reaction (PCR) and sequencing.

### 2.3 Primer extension

Primer extension analyses of the rRNAs were used to determine whether the *tlyA* ^*I*^‐type and *tlyA* ^*II*^ gene products were expressed and retained their activity. RNA was prepared from *C. jejuni* as described previously by Douthwaite, Powers, Lee, and Noller (1989). 5′‐^32^P‐end‐labeled deoxynucleotide primers were hybridised to complementary regions of 16S rRNA nucleotides 1411–1429 (primer 5′‐GTGAAATCAACTCCCATGG) and 23S nucleotides 1924–1941 (primer 5′‐GAATTTCGCTACCTTAGG); *Escherichia coli* rRNA numbering is used throughout. Primers were extended with AMV reverse transcriptase (Roche), and the extension products were run on denaturing polyacrylamide/urea gels to detect sites of 2′‐*O*‐methylation (Johansen et al., 2006; Maden, Corbett, Heeney, Pugh, & Ajuh, 1995).

### 2.4 Minimal inhibitory concentration (MIC) determination

Overnight cultures of the *C. jejuni* strains were diluted to a turbidity of 0.5 McFarland standard, and 3 μl were plated onto BHI agar plates with two‐fold increases in the capreomycin concentration. The MIC values are the lowest concentration of antibiotic at which no growth was observed after incubation under microaerobic conditions for 48 hr at 37°C.

### 2.5 Motility

The motility of *C. jejuni* cells was assessed by adding 3 μl of culture (OD_600_ 0.5) onto BHI with 0.25% agar. Plates were left to dry and were incubated under microaerobic conditions for 48 hr at 37°C before measuring cell migration.

### 2.6 Biofilm assays

Three‐dimension confocal microscope images of biofilms were produced from *C. jejuni* grown on glass slides (Millicell EZ, Milllipore). Strains were diluted in BHI broth to OD_600_ 0.05 before aliquoting to the Millicell dishes and incubating at 37°C for 48 hr. Broth was removed, and biofilms were washed twice with water and dried at 55°C for 15 min before staining with acridine orange solution (1 μg/ml) for 30 min and rinsing twice with PBS. Biofilms were visualised at an excitation wavelength of 490 nm using a Leica white laser scanning confocal microscope (Leica TCS SP8‐WWL) with a 63× oil‐immersion lens. Three‐dimensional images were created from Z‐stacks images collected from top down to obtain an overall view of the biofilm volume and converted to TIFF files with depth‐coding using LAS X software (Leica Microsystems).

### 2.7 Field Emission Scanning Electron Microscopy

Visualisation of cell morphology was carried out using field emission scanning electron microscopy (FESEM). *C. jejuni* cells were grown on Columbia agar plates for 24 hr before harvesting and suspending in 5 ml BHI broth (at OD600 of 0.05) and cultivating for 48 hr at 37°C in 5% CO_2_ on glass cover slides. Cells were fixed for 24 hr in 0.1 M cacodylate buffer (pH 7.3) with 3% glutaraldehyde followed by washing for 60 min in cacodylate buffer without glutaraldehyde, and then four times for 30 min in fresh buffer followed by dehydration for 6 hr in 96% ethanol. Cells were air dried and coated with gold–palladium (2–4 nm thick) and analysed at nanometer image resolution by FESEM (MERLIN Carl Zeiss Germany) at 2–5 kV range accelerating voltage.

### 2.8 Adhesion and invasion assays

Caco‐2 epithelial cells, derived from a human colonic carcinoma, were seeded into a 24‐well tissue culture dishes and grown overnight at 37°C to a cell density of 10^5^ cells per well in Eagle's minimum essential medium containing Earle's salts, 2 mM l‐glutamine, 10% fetal bovine serum, 0.1 mM nonessential amino acids and 1 mM sodium pyruvate in a 5% CO_2_ (CO_2_ incubator, Thermo Scientific).

The *C. jejuni* strains were added into the wells at a multiplicity of infection (MOI) of one hundred bacteria to one epithelial cell and incubated for 2 hr to allow adhesion and invasion of the Caco‐2 cells. The Caco‐2 monolayers were washed three times with PBS to remove unattached bacteria. A portion of the Caco‐2 cells was then lysed with 0.1% Triton X‐100 to estimate the total complement of bacterial cells. The remaining Caco‐2 cells were incubated for a further 2 hr in modified minimal essential medium with 100 μg gentamicin ml^−1^ to kill extracellular bacteria, while retaining viable internalised bacteria. Bacteria adhering to and internalised by the Caco‐2 cells were tallied by serial dilution in phosphate‐buffered saline (PBS) and plating on BHI agar.

### 2.9 Survival assay

*C. jejuni* survival was quantified in RAW 264.7 macrophages cultured in RPMI medium with 10% fetal bovine serum at 37°C in 5% CO_2_ atmosphere (CO_2_ incubator, Thermo Scientific). Tissue culture trays (24‐well) were seeded with 2 × 10^5^ macrophages per ml and incubated for 24 hr prior to inoculating with *C. jejuni* at an approximate MOI of 100. Infected macrophage monolayers were incubated for 2 hr before killing extracellular bacteria as described above. Surviving bacteria were monitored (as above) at 3, 6, 12, 24 and 48 hr post‐infection.

### 2.10 Innate immune response in epithelial cells

Production of interleukin of IL‐8 by Caco‐2 cells was taken as an indicator of the extent to which by *C. jejuni* strains provoked an innate immune response. Caco‐2 cells were seeded in 24‐well plates and infected with bacteria as described above. Cell supernatants were assayed after one day using a human IL‐8 ELISA kit (Merck). Optical densities were measure with a microplate reader (Epoch spectrophotometer, BioTek Instruments) and normalised relative to negative controls (no *C. jejuni* cells) using the instrument supplier's software.

## 3 RESULTS

### 3.1 *Mycobacterial* TlyA^II^ specifically methylates two nucleotides in *C. jejuni* rRNAs

**Figure 1:**
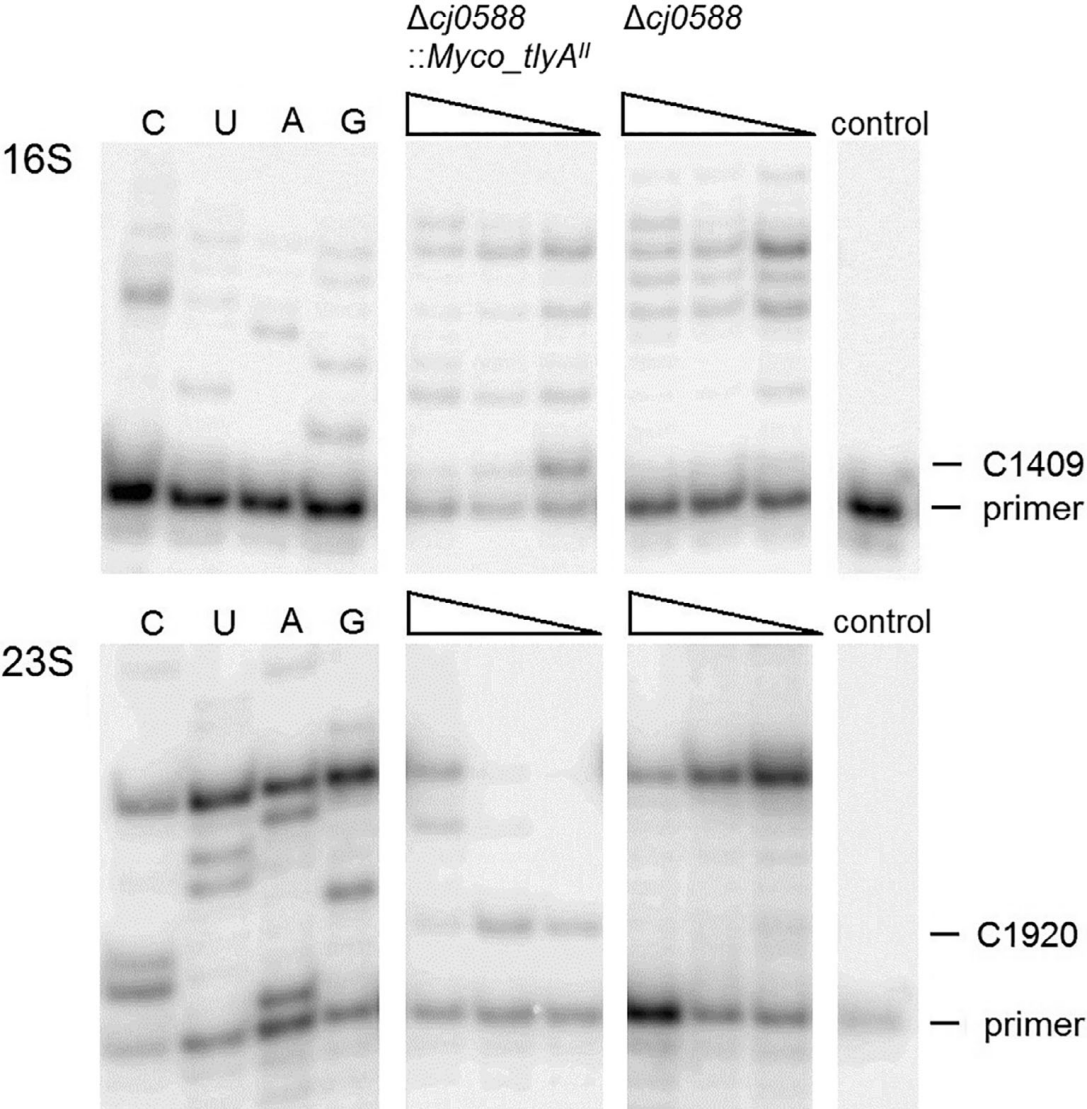
*In vivo* activity of mycobacterial TlyA^II^ in *C. jejuni*. Gel autoradiograms of primer extensions on rRNA from *C. jejuni* strains. Extensions on 16S and 23S rRNAs from the mutant strain *C. jejuni* 81–176 Δ*cj0588* and from the same strain complemented with mycobacterial *tlyA* ^*II*^ (*C. jejuni* 81–176 Δ*cj0588*::*Myco_tlyA* ^*II*^). Decreasing the dGTP concentrations (100, 10 and 1 μM, marked with wedges) intensifies reverse transcription termination at 16S rRNA C1409 and 23S rRNA C1920 when these nucleotides are 2′‐*O*‐methylated. Lanes C, U, A and G are dideoxy‐sequencing reactions on unmodified *C. jejuni* rRNAs. Control lanes represent primers and reaction mixture without rRNA template

The wild type *tlyA* ^*II*^ gene from *M. smegmatis* was introduced into the *C. jejuni* null strain 81‐176Δ*cj0588*. Screening the rRNAs from this recombinant by primer extension showed that expression of the mycobacterial TlyA^II^ enzyme effectively modified nucleotide C1409 in *C. jejuni* 16S rRNA and C1920 in the 23S rRNA (*E. coli* rRNA numbering) (Figure 1).

These modifications resulted in a concomitant increase in the sensitivity of *C. jejuni* to capreomycin. The MIC values for capreomycin in strains without a *tlyA* gene (81176Δ*cj0588*) or with an inactivated version of the gene (81176Δ*cj0588*::K188A) were consistently 64 μg/ml. The capreomycin MIC was lowered to 32 μg/ml by expression of the mycobacterial *tlyA* ^*II*^ gene that was the same value seen for cells expressing the original *tlyA* ^*I*^ gene *cj0588* that methylates only at 23S rRNA nucleotide C1920.

### 3.2 Motility and rRNA methylation

**Figure 2:**
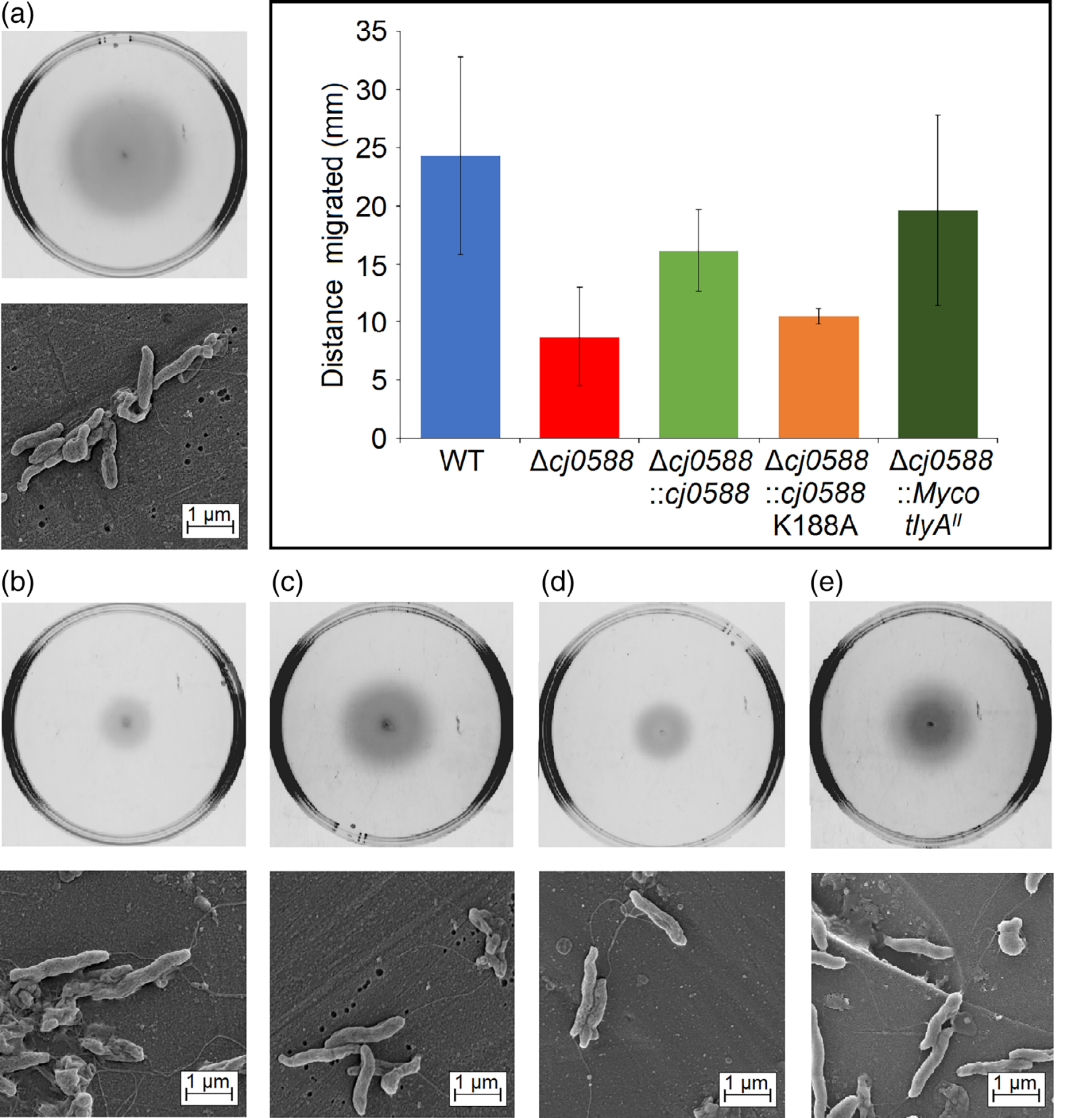
Motility of the *C. jejuni* strains. Agar plates with (a) WT‐wild type strain, (b) Δ*cj0588*, (c) Δ*cj0588*::*cj0588*, (d) Δ*cj0588*::*cj0588*K188A and (e) Δ*cj0588*::*Myco_tlyA* ^*II*^ strains of *C. jejuni* grown for 48 hr. The morphologies of the corresponding stains (including flagella) were visualised by Field Emission Scanning Electron Microscopy (FESEM). Strain motility is summarised in the histogram, where values represent the means ± *SEM* of three independent experiments measuring distances migrated over 48 hr. There was no significant difference for migration of the WT compared to Δ*cj0588*::*M.smeg_tlyA* ^*II*^, whereas significant differences were observed for Δ*cj0588* versus Δ*cj0588*::*M.smeg_tlyA* ^*II*^ (*p* < .05), and for Δ*cj0588* versus Δ*cj0588*::*cj0588* (*p* < .05)

Loss or inactivation of its natural *tlyA* ^*I*^ gene results in decreased *C. jejuni* motility. Under the microaerobic conditions at 37°C employed here, *C. jejuni* null strains exhibit only one‐third of the mobility of the wild‐type (Figure 2). Complementation with an active copy of *cj0588* goes some way to restoring mobility to wild‐type levels. Similarly, introduction of the mycobacterial *tlyA* ^*II*^ gene into the null strain partially rescues the cell's motility (Figure 2).

### 3.3 Biofilm formation is influenced by the rRNA methylation pattern

**Figure 3:**
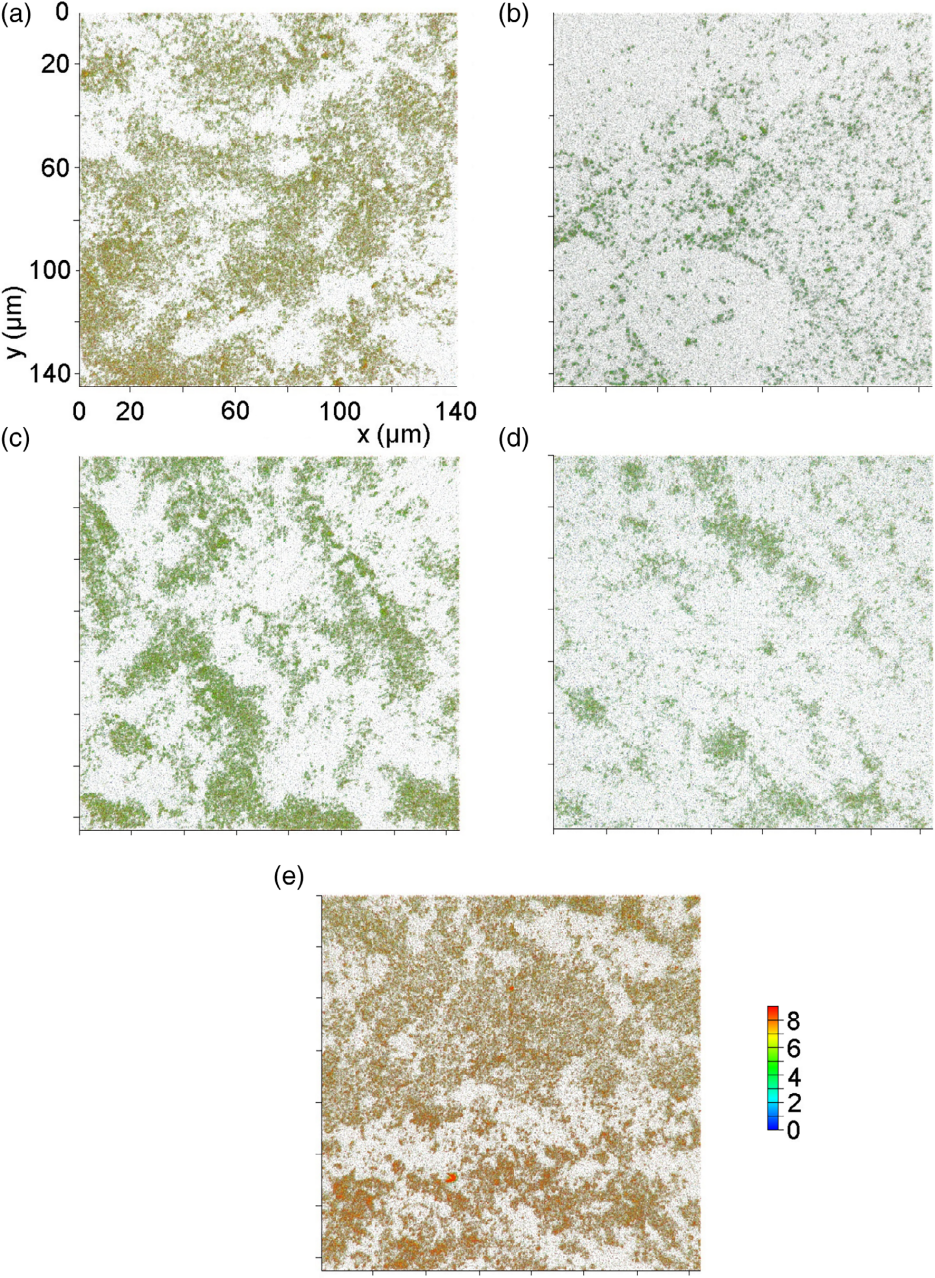
Images of biofilm structures produced by *C. jejuni* (a) WT‐wild type strain, (b) Δ*cj0588*, (c) Δ*cj0588*::*cj0588*, (d) Δ*cj0588*::*cj0588*K188A and (e) Δ*cj0588*::*Myco_tlyA* ^*II*^ strains visualised by confocal laser microscopy. Relative biofilm depths are color‐coded as shown

**Figure 4:**
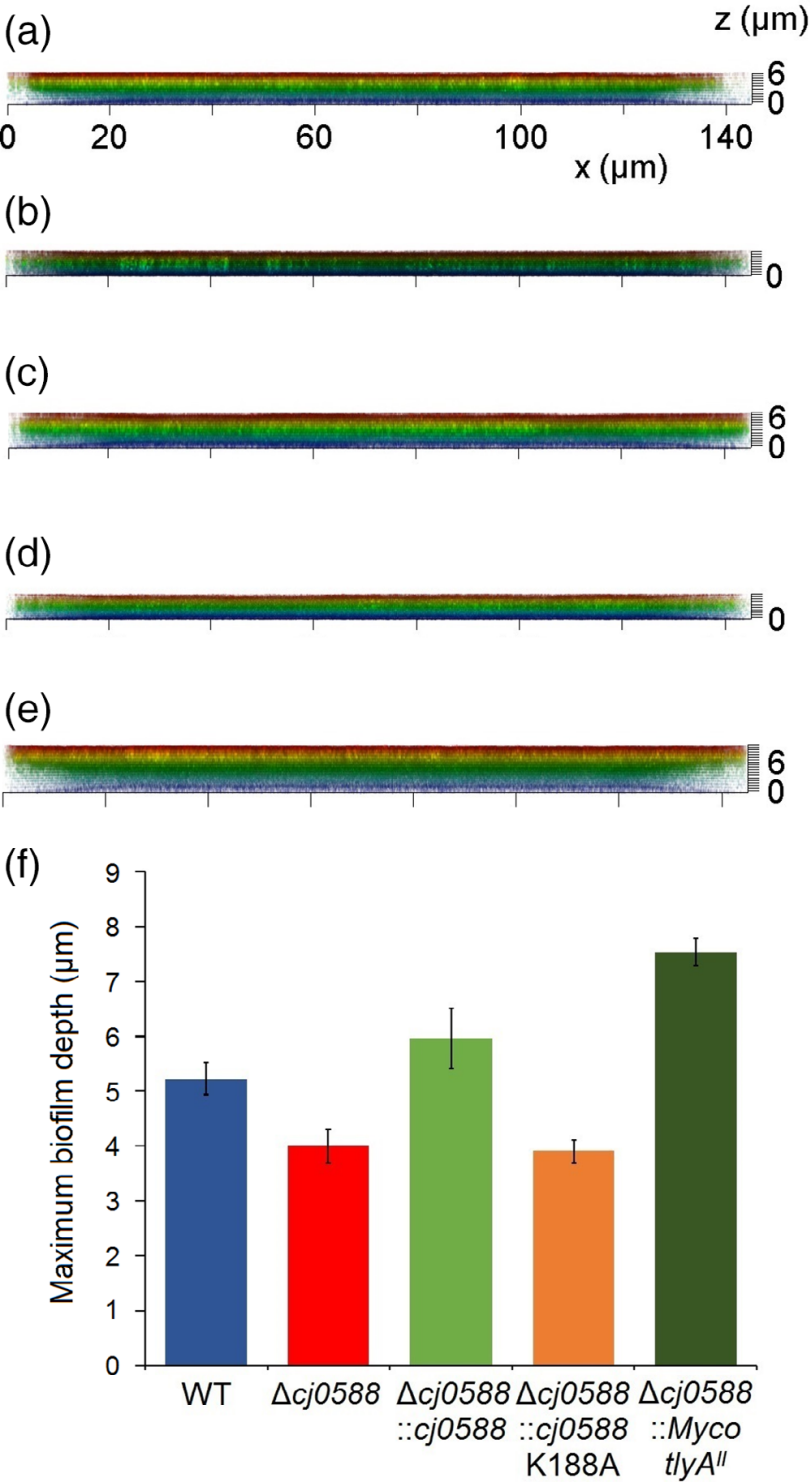
Analysis of the Figure 3 images showing the biofilm density produced by *C. jejuni* (a) WT‐wild type strain, (b) Δ*cj0588*, (c) Δ*cj0588*::*cj0588*, (d) Δ*cj0588*::*cj0588*K188A and (e) Δ*cj0588*::*Myco_tlyA* ^*II*^ strains. (f) Histogram summarising the biofilm data, color‐coded as in Figure 2. Experiments were carried out in triplicate and representative images are shown here. *p* < .001 for WT versus Δ*cj0588*; *p* < .005 for WT versus Δ*cj0588*::*M.smeg_tlyA* ^*II*^

Biofilms were visualised using confocal laser microscopy producing three‐dimensional images of these structures (Figures 3 and 4). Inactivation of the *cj0588* gene reduces the cell's ability to form biofilms, and this effect is rescued by introduction of a functional copy of this gene. The null strain formed a thin biofilm that failed cover the whole surface and reached a depth of only 3.9 μm in its thickest region. Complementation with an active *cj0588* gene fully restored biofilm density to 5.9 μm, comparable to that of the wild‐type strain (5.2 μm). Surprisingly, transformation of the null strain with the mycobacterial *tlyA* ^*II*^ gene (forming the 81176Δ*cj0588*::*Myco_tlyA* ^*II*^ strain) not only rescued the phenotype but supported uniform biofilm formation at a density of 7.5 μm, significantly surpassing that the wild‐type strain (Figure 4).

### 3.4 Adhesion and invasion of the *C. jejuni* strains on Caco‐2 cells

**Figure 5:**
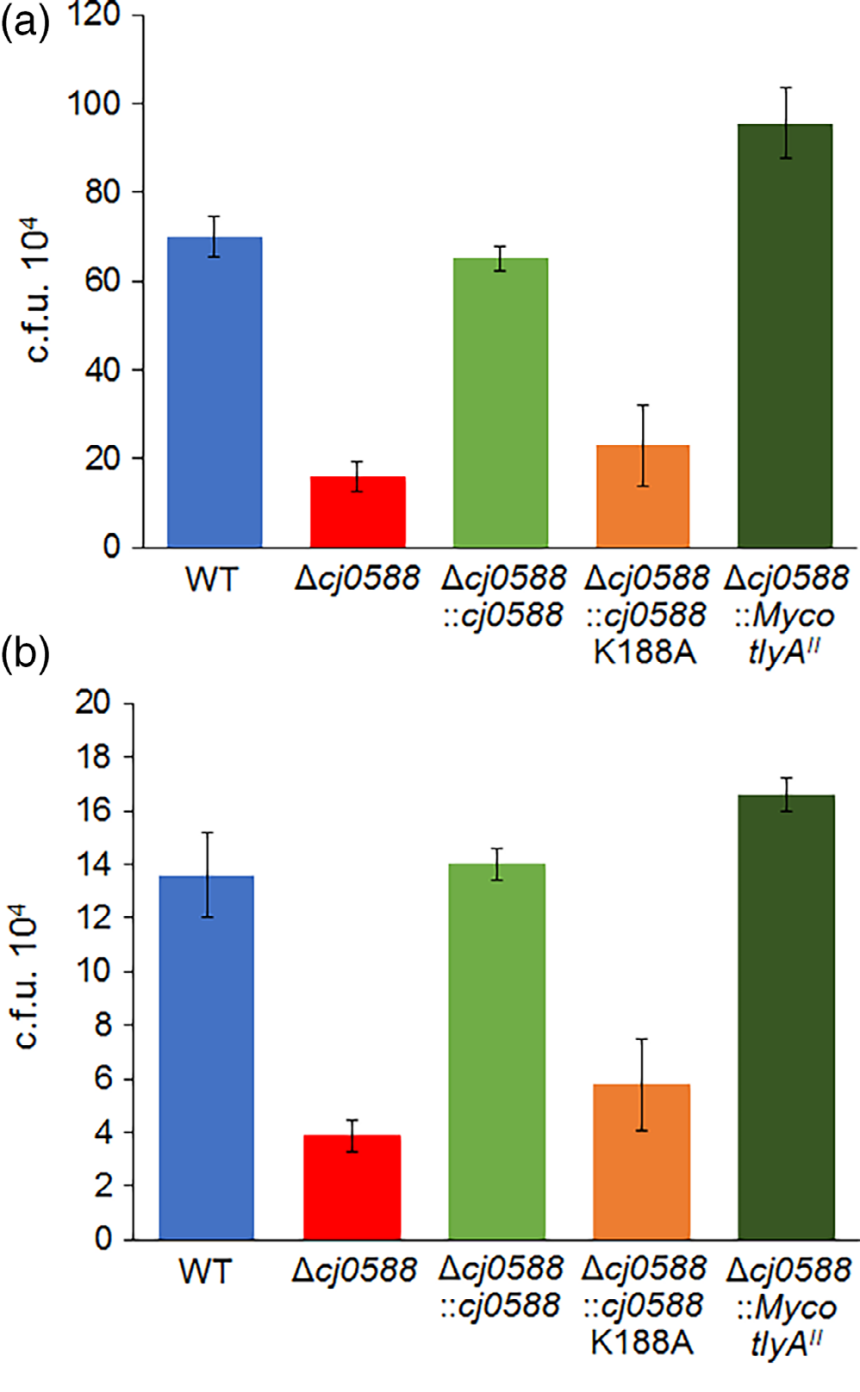
(a) Adhesion onto, and (b) invasion into Caco‐2 cells by *C. jejuni* strains. Values represent means ± *SEM* of three independent experiments. Adhesion *p* < .05 for WT versus Δ*cj0588* and Δ*cj0588*::*Myco_tlyA* ^*II*^; invasion *p* < .005 for WT versus Δ*cj0588* and Δ*cj0588*::*Myco_tlyA* ^*II*^

After inactivation of TlyA‐directed methylation, the capacity of *C. jejuni* to adhere to the surface and to invade Caco‐2 human colon epithelial cells was reduced to less than half that of the wild‐type (Figure 5). Both adhesion and internalisation were restored (to 93 and 103% wild‐type levels, respectively) by complementation of the null strain with *cj0588*. This effect was more marked after complementation with the mycobacterial gene where the *C. jejuni tlyA* ^*II*^ recombinants became about one‐third more adept than the wild‐type at sticking to Caco‐2 cells enabling the pathogen to invade the epithelial cells 25% more effectively (Figure 5). The bacterial strains' ability to enter the Caco‐2 cells was roughly proportional to their adhesive properties and thus the invasive index, which is the proportion of the surface‐adhered bacteria that actually enter the eukaryotic cell, was fairly constant (i.e., varied less than 25%) for the different *C. jejuni* recombinants.

**Figure 6:**
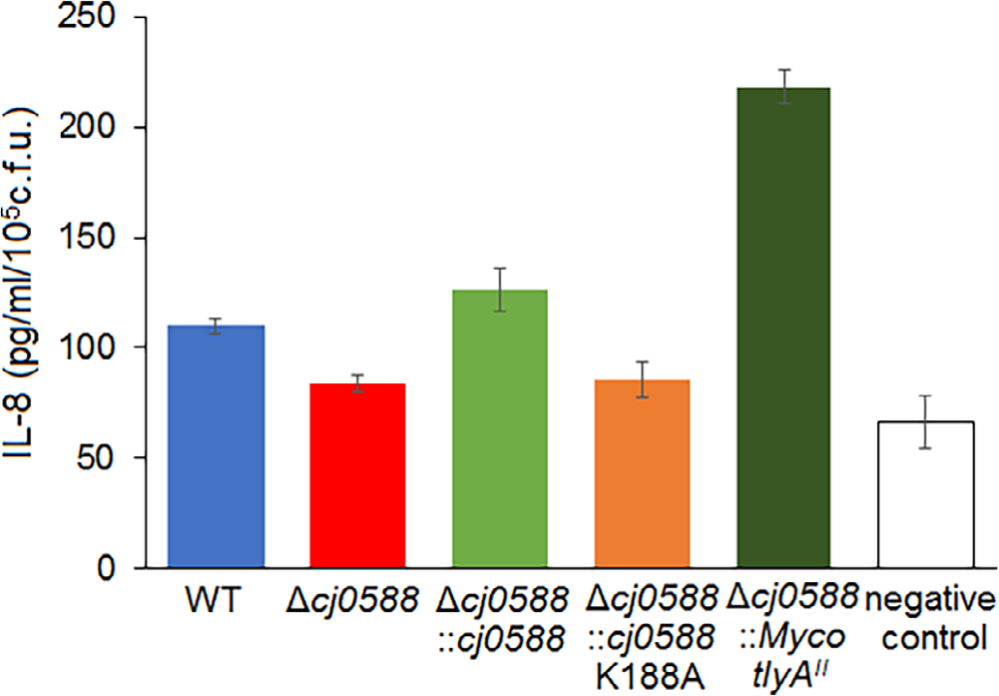
IL‐8 secretion by Caco‐2 induced by *C. jejuni* strains. The negative control shows the background level of IL‐8 secretion measured in the absence of *C. jejuni* cells. Values represent means ± *SEM* of three independent experiments. *p* < .05 for WT versus Δ*cj0588*; *p* < .01 for WT versus Δ*cj0588*::*Myco_tlyA* ^*II*^

Subsequent to invasion, the virulence of the *C. jejuni* attack was inferred from the IL‐8 response within the Caco‐2 epithelial cell line. Consistent with the adhesion/invasion data, the mildest reaction was seen with the *cj0588*‐deletion strain and the inactive K188A variant, where IL‐8 levels were barely above background (Figure 6). The wild‐type and complemented strains with an active cj0588 gene produced a clearer response, approximately doubling the amount of IL‐8. The highest level of IL‐8 was observed with *C. jejuni* expressing the mycobacterial *tlyA* ^*II*^ gene, reflecting the augmented adhesion/invasion properties of this strain in the Caco‐2 cell model.

### 3.5 Influence of the rRNA methylation pattern on bacterial survival in macrophages

Another important aspect of *C. jejuni* virulence is its ability to survive within host cells, and this was tested here using a RAW264.7 macrophage model system. While *C. jejuni* strains expressing functional TlyA proteins attached to and invaded the macrophages significantly more avidly than null strains, their survival within macrophages was not improved (Table 2), and all the *C. jejuni* strains within macrophages were killed by 48 hr.

**Table 2:** Survival of *C. jejuni* strains within macrophages

Hours after infection	Colony forming units (cfu) of *C. jejuni strains surviving*				
	WT	Δ*cj0588*	Δ*cj0588*::*cj0588*	Δ*cj0588*::*cj0588* K188A	Δ*cj058*8::*Myco tlyA* ^*II*^
3	281,035 ± 29,856	45,886 ± 30,052	274,700 ± 69,296	23,669 ± 10,394	284,701 ± 69,296
6	142,570 ± 3,110	35,375 ± 7,443	155,000 ± 34,156	14,525 ± 2,227	92,600 ± 10,465
12	3,002 ± 87	1,268 ± 381	3,446 ± 702	481 ± 99	2,713 ± 636
24	300 ± 36	125 ± 77	200 ± 13	35 ± 23	36 ± 23
48	0	0	0	0	0

*Note*: Macrophage RAW264.7 cell line samples were each infected with 10^7^ cfu of the various *C. jejuni* strains (time zero). Viable intracellular *C. jejuni* cells are tabulated with shading to indicate >200,000; 50,000 to 200,000; 2,000 to 50,000; 200 to 2,000; and 20 to 200 surviving cells over 24 hr. No viable *C. jejuni* cells were detected at 48 hr. The cfu values are means ± *SEM* of three independent experiments; *p* < .05 for WT versus Δ*cj0588*; *p* < .01 for WT versus Δ*cj0588*::*Myco_tlyA* ^*II*^.

## 4 DISCUSSION

The natural version of the TlyA methyltransferase found in *C. jejuni* is a type I (TlyA^I^) enzyme encoded by the *cj0588* gene, and stoichiometrically methylates the 2´‐*O*‐ribose of 23S rRNA nucleotide C1920 (Sałamaszyńska‐Guz et al., 2018). Type II (TlyA^II^) variants, found in some Gram‐positive bacteria including the Actinobacteria, methylate not only at nucleotide C1920 but also at 16S rRNA nucleotide C1409 (Johansen et al., 2006; Monshupanee et al., 2012). Both these nucleotides are effectively modified in the natural *M. smegmatis* host (Monshupanee et al., 2012), and the mycobacterial *tlyA* ^*II*^ gene product retains the same specificity when transferred to and expressed from the *C. jejuni* chromosome (Figure 1).

We have previously shown that a possession of an active TlyA^I^ (Cj0588) methyltransferase is a prerequisite for *C. jejuni* to function effectively as a pathogen (Sałamaszyńska‐Guz & Klimuszko, 2008; Sałamaszyńska‐Guz et al., 2014). The role of TlyA^I^ in pathogenicity is linked to a series of factors including ribosomal subunit interaction, cell motility and biofilm formation, all of which were depressed in *cj0588* null strains (Sałamaszyńska‐Guz et al., 2018). These properties are rescued by complementation with an active copy of *cj0588*, although no rescue occurs after eliminating the catalytic activity of the enzyme by introducing point mutations into *cj0588*. These methyltransferase mutants retained their tertiary structure and their substrate/cofactor binding affinities, and thus the reduction in *C. jejuni* virulence was solely a consequence of the mutant enzymes' inability to methylate the rRNA (Sałamaszyńska‐Guz et al., 2018).

Homologs of this enzyme have also been linked to virulence in other bacterial pathogens. Mutation of the *tlyA* ^*I*^ homolog in *B. hyodysenteriae* reduces virulence (Hyatt et al., 1994), while loss of the enzyme's function in *H. pylori* lowers adhesion to human gastric adenocarcinoma (AGS) cells and prevents colonisation of the gastric mucosa (Martino et al., 2001; Zhang et al., 2002). The TlyA^II^ variant of this enzyme promotes survival of *M. tuberculosis* in macrophages (Rahman et al., 2015) and aids the binding of capreomycin to ribosomes (Maus et al., 2005). Several other endogenous rRNA methylations (reviewed in Purta, O'Connor, Bujnicki, & Douthwaite, 2009) have also been noted to promote ribosome–antibiotic interactions within bacterial pathogens (LaMarre, Howden, & Mankin, 2011; Sergeeva, Bogdanov, & Sergiev, 2015). In the present study, we demonstrate that the defective virulence traits exhibited in *C. jejuni tlyA* null strains can be rescued by a mycobacterial *tlyA* ^*II*^ ortholog, and that some phenotypic traits of the recombinant strain are distinctly different from the original wild‐type and recombinants rescued with the original *cj0588 (tlyA* ^*I*^ *)* gene.

The lower mobility of the *C. jejuni* null strain was restored to roughly the same extent by the wild‐type *C. jejuni tlyA* ^*I*^ gene and the mycobacterial *tlyA* ^*II*^ gene (Figure 2). However, the recombinants differed in their capacity to form biofilms. Complementation with *tlyA* ^*I*^ re‐establishes wild‐type levels, while cells transformed with *tlyA* ^*II*^ form significantly denser biofilms (Figures 3 and 4). Visualisation of the cells using FESEM revealed that the cell morphology and flagella structure of the *tlyA* ^*I*^ (Sałamaszyńska‐Guz et al., 2018) and the *tlyA* ^*II*^ recombinants (Figure 2) were indistinguishable from that of wild‐type *C. jejuni* cells. Surprisingly, therefore, loss of TlyA function does not affect flagella morphology despite the central role of flagella in *C. jejuni* pathogenesis (Guerry, 2007; Svensson, Pryjma, & Gaynor, 2014).

The *tlyA* ^*II*^ homolog of *M. tuberculosis*, which is 84% similar in amino acid sequence and functionally identical to the *M. smegatis tlyA* ^*II*^ (Monshupanee et al., 2012), plays an important role in the survival of the bacillus during infection (Rahman et al., 2015). *M. tuberculosis* cells lacking *tlyA* ^*II*^ become more susceptible to autophagy, and animals infected with this mutant strain exhibit increased immune response, reduced bacillary load and improved survival rates than when infected with wild‐type bacilli (Rahman et al., 2015). Our findings here suggest that the mycobacterial *tlyA* ^*II*^ gene supports a comparable set of virulence traits in *C. jejuni*.

The ability of *C. jejuni* to attach to and invade human epithelial cells is central to its pathogenicity and was notably impaired by loss of TlyA‐directed methylation (Figure 5). Restoring TlyA^I^ methylation by complementation of *C. jejuni* with *cj0588* rescued its adhesion to and invasion of Caco‐2 cells. Surprisingly, these features were not only rescued by the mycobacterial *tlyA* ^*II*^ but this recombinant clung to and entered the epithelial cells significantly more effectively than the original wild‐type strain *C. jejuni* (Figure 5).

When under attack by pathogenic bacteria, epithelial cells secrete chemotactic mediators (Eckmann, Kagnoff, & Fierer, 1993) and consistent with this, *C. jejuni* induces human‐derived epithelial cell lines to release pro‐inflammatory chemokines including the interleukin, IL‐8 (Hickey, Baqar, Bourgeois, Ewing, & Guerry, 1999; Watson & Galan, 2005). In related studies of *Campylobacter* invasion, cytolethal distending toxin, outer membrane vesicles and the flagella activate the host cell's toll‐like receptors to elicit secretion of IL‐8 (Zheng, Meng, Zhao, Singh, & Song, 2008). The absence of *tlyA* activity is shown here to reduce the ability of *C. jejuni* to trigger the IL‐8 innate immune response in Caco‐2 cells (Figure 6). Induction of IL‐8 was restored by complementing null strains with an active *tlyA* gene, where the *tlyA* ^*II*^ gene produced the most marked stimulation increasing IL‐8 production to twice that with wild‐type *C. jejuni*.

These observations raise a number of questions. First, why a single ribose methylation (at 23S rRNA nucleotide C1920) would be a prerequisite for successful infection by *C. jejuni* and how an additional methylation (at 16S rRNA nucleotide C1409) would further improve its capacity to infect. The two TlyA^II^ methylations are located approximately 20 Å apart on opposite sides of the ribosomal subunit interface and lie adjacent to the capreomycin binding site (Johansen et al., 2006). Both methylations have been shown to contribute individually to drug binding (Monshupanee et al., 2012). From the crystal structure of capreomycin‐bound ribosomes (Stanley et al., 2010), the methylations are slightly too far apart to make contact with the drug. However, they are nevertheless positioned where they might lubricate the relative rotational movement of the subunits during translation (Yusupov et al., 2001), a process where one of the pivoted subunit conformations is favoured for drug binding (Ermolenko et al., 2007). Each of the methylations thus contributes to ribosome function, and our working hypothesis (presently being tested) is that changes in the methylation pattern subtly skew the relative synthesis rates of different proteins in the bacterium, with this being ultimately reflected in altered virulence properties.

Another, and potentially more important, question is whether it would make a difference in the real world if *C. jejuni* were to attain both methylations through changes in its own *tlyA* gene or via transfer of a *tlyA* ^*II*^ ortholog from another bacterium. The ability of *C. jejuni* to adhere to epithelial cells is dependent on having a functional *tlyA* gene and is enhanced with a *tlyA* ^*II*^‐type gene, and these adhesive properties determine the degree of cell invasion (Figure 5). An additional aspect to be taken into consideration is that *C. jejuni* pathogenicity depends on its ability to survive subsequent to phagocytosis. On the one hand, the RAW 264.7 macrophage data (Table 2) show that significantly fewer *C. jejuni* survive when they lack an active *cj0588* gene and that complementing the cells with an active copy of *cj0588* or the mycobacterial *tlyA* ^*II*^ gene restores their initial survival rates. This observation is consistent with the role of *tlyA* ^*II*^ mentioned above, where it supports the survival within macrophages of its authentic host, *M. tuberculosis* (Rahman et al., 2015). However, when extending the time frame of observations past the initial phagocytotic event, we find that the *C. jejuni*‐*tlyA* ^*II*^ cells are no more resilient after 12 hours (and in fact appear slightly more frail) than strains expressing *cj0588* (Table 2). In this case, the wild‐type *cj0588* gene affords better protection against the host's defences, and it thus remains an open question to what extent the superior adhesion properties conferred by *tlyA* ^*II*^ represent a route towards increased *C. jejuni* virulence.

## CONFLICT OF INTEREST

The authors have no conflicts of interest to declare.

## References

[cmi13199-bib-0001] Douthwaite, S. , Powers, T. , Lee, J. Y. , & Noller, H. F. (1989). Defining the structural requirements for a helix in 23S ribosomal RNA that confers erythromycin resistance. Journal of Molecular Biology, 209(4), 655–665. 2685326 10.1016/0022-2836(89)93000-3

[cmi13199-bib-0002] Eckmann, L. , Kagnoff, M. F. , & Fierer, J. (1993). Epithelial cells secrete the chemokine interleukin‐8 in response to bacterial entry. Infection and Immunity, 61(11), 4569–4574. 8406853 10.1128/iai.61.11.4569-4574.1993 PMC281206

[cmi13199-bib-0003] Ermolenko, D. N. , Spiegel, P. C. , Majumdar, Z. K. , Hickerson, R. P. , Clegg, R. M. , & Noller, H. F. (2007). The antibiotic viomycin traps the ribosome in an intermediate state of translocation. Nature Structural & Molecular Biology, 14(6), 493–497. 10.1038/nsmb1243 17515906

[cmi13199-bib-0004] Guerry, P. (2007). Campylobacter flagella: Not just for motility. Trends in Microbiology, 15, 456–461. 17920274 10.1016/j.tim.2007.09.006

[cmi13199-bib-0005] Hickey, T. E. , Baqar, S. , Bourgeois, L. , Ewing, C. P. , & Guerry, P. (1999). *Campylobacter jejuni* stimulated secretion of interleukin‐8 by INT407 cells. Infection and Immunity, 67(1), 88–93. 9864200 10.1128/iai.67.1.88-93.1999 PMC96281

[cmi13199-bib-0006] Hyatt, D. R. , ter Huurne, A. A. , van der Zeijst, B. A. , & Joens, L. A. (1994). Reduced virulence of *Serpulina hyodysenteriae* hemolysin‐negative mutants in pigs and their potential to protect pigs against challenge with a virulent strain. Infection and Immunity, 62(6), 2244–2248. 8188345 10.1128/iai.62.6.2244-2248.1994 PMC186504

[cmi13199-bib-0007] Javed, M. A. , Cawthraw, S. A. , Baig, A. , Li, J. , McNally, A. , Oldfield, N. J. , … Manning, G. (2012). Cj1136 is required for lipooligosaccharide biosynthesis, hyperinvasion, and chick colonization by *Campylobacter jejuni* . Infection and Immunity, 80(7), 2361–2370. 22508861 10.1128/IAI.00151-12 PMC3416457

[cmi13199-bib-0008] Johansen, S. K. , Maus, C. E. , Plikaytis, B. B. , & Douthwaite, S. (2006). Capreomycin binds across the ribosomal subunit interface using *tlyA*‐encoded 2′‐O‐methylations in 16S and 23S rRNAs. Molecular Cell, 23(2), 173–182. 16857584 10.1016/j.molcel.2006.05.044

[cmi13199-bib-0009] Korlath, J. A. , Osterholm, M. T. , Judy, L. A. , Forfang, J. C. , & Robinson, R. A. (1985). A point‐source outbreak of campylobacteriosis associated with consumption of raw milk. Journal of Infectious Diseases, 152(3), 592–596. 4031557 10.1093/infdis/152.3.592

[cmi13199-bib-0010] LaMarre, J. M. , Howden, B. P. , & Mankin, A. S. (2011). Inactivation of the indigenous methyltransferase RlmN in *Staphylococcus aureus* increases linezolid resistance. Antimicrob Agents and Chemotherapy, 55(6), 2989–2991. 10.1128/AAC.00183-11 PMC3101465 21444696

[cmi13199-bib-0011] Maden, B. E. H. , Corbett, M. E. , Heeney, P. A. , Pugh, K. , & Ajuh, P. M. (1995). Classical and novel approaches to the detection and localization of the numerous modified nucleotides in eukaryotic ribosomal RNA. Biochimie, 77(1–2), 22–29. 7599273 10.1016/0300-9084(96)88100-4

[cmi13199-bib-0012] Martino, M. C. , Stabler, R. A. , Zhang, Z. W. , Farthing, J. G. , Wren, B. W. , & Dorrell, N. (2001). *Helicobacter pylori* pore‐forming orthologue TlyA possesses in vitro hemolytic activity and has a role in colonization of gastric mucosa. Infection and Immunity, 69(3), 1697–1703. 11179345 10.1128/IAI.69.3.1697-1703.2001 PMC98074

[cmi13199-bib-0013] Maus, C. E. , Plikaytis, B. B. , & Shinnick, T. M. (2005). Mutation of *tlyA* confers capreomycin resistance in *Mycobacterium tuberculosis* . Antimicrobial Agents and Chemotherapy, 49(2), 571–577. 15673735 10.1128/AAC.49.2.571-577.2005 PMC547314

[cmi13199-bib-0014] Monshupanee, T. (2013). Increased bacterial hemolytic activity is conferred by expression of TlyA methyltransferase but not by its 2′‐*O*‐methylation of the ribosome. Current Microbiology, 67, 61–68. 23417025 10.1007/s00284-013-0332-7

[cmi13199-bib-0015] Monshupanee, T. , Johansen, S. K. , Dahlberg, A. E. , & Douthwaite, S. (2012). Capreomycin susceptibility is increased by TlyA‐directed 2′‐*O*‐methylation on both ribosomal subunits. Molecular Microbiology, 85(6), 1194–1203. 22779429 10.1111/j.1365-2958.2012.08168.x PMC3438285

[cmi13199-bib-0016] Purta, E. , O'Connor, M. , Bujnicki, J. M. , & Douthwaite, S. (2009). YgdE is the 2′‐O‐ribose methyltransferase RlmM specific for nucleotide C2498 in bacterial 23S rRNA. Molecular Microbiology, 72, 1147–1158. 19400805 10.1111/j.1365-2958.2009.06709.x

[cmi13199-bib-0017] Rahman, M. A. , Sobia, P. , Dwivedi, V. P. , Bhawsar, A. , Singh, D. K. , Sharma, P. , … Das, G. (2015). *Mycobacterium tuberculosis* TlyA negatively regulates Th1 and Th17 differentiation and promotes tuberculosis pathogenesis. Journal of Biological Chemistry, 5(23), 14407–14417. 10.1074/jbc.M115.653600 PMC4505508 25847237

[cmi13199-bib-0018] Sałamaszyńska‐Guz, A. , Grodzik, M. , & Klimuszko, D. (2013). Mutational analysis of *cj0183 Campylobacter jejuni* promoter. Current Microbiology, 67(6), 696–702. 23884593 10.1007/s00284-013-0420-8 PMC3824568

[cmi13199-bib-0019] Sałamaszyńska‐Guz, A. , & Klimuszko, D. (2008). Functional analysis of the *Campylobacter jejuni cj0183* and *cj0588* genes. Current Microbiology, 56(6), 592–596. 10.1007/s00284-008-9130-z 18389311

[cmi13199-bib-0020] Sałamaszyńska‐Guz, A. , Rose, S. , Lykkebo, C. A. , Taciak, B. , Bącal, P. , Uśpieński, T. , & Douthwaite, S. (2018). Biofilm Formation and Motility Are Promoted by Cj0588‐Directed Methylation of rRNA in *Campylobacter jejuni* . Frontiers in Cellular and Infection Microbiology, 7, 533 10.3389/fcimb.2017.00533 29404277 PMC5778110

[cmi13199-bib-0021] Sałamaszyńska‐Guz, A. , Taciak, B. , Kwiatek, A. , & Klimuszko, D. (2014). The Cj0588 protein is a *Campylobacter jejuni* RNA methyltransferase. Biochemical and Biophysical Research Communications, 448(3), 298–302. 24796671 10.1016/j.bbrc.2014.04.104

[cmi13199-bib-0022] Sergeeva, O. V. , Bogdanov, A. A. , & Sergiev, P. V. (2015). What do we know about ribosomal RNA methylation in *Escherichia coli*? Biochimie, 117, 110–118. 25511423 10.1016/j.biochi.2014.11.019

[cmi13199-bib-0023] Stanley, R. E. , Blaha, G. , Grodzicki, R. L. , Strickler, M. D. , & Steitz, T. A. (2010). The structures of the anti‐tuberculosis antibiotics viomycin and capreomycin bound to the 70S ribosome. Nature Structural & Molecular Biology, 17(3), 289–293. 10.1038/nsmb.1755 PMC2917106 20154709

[cmi13199-bib-0024] Svensson, S. L. , Pryjma, M. , & Gaynor, E. C. (2014). Flagella‐mediated adhesion and extracellular DNA release contribute to biofilm formation and stress tolerance of *Campylobacter jejuni* . PLoS One, 9, e106063. 25166748 10.1371/journal.pone.0106063 PMC4148357

[cmi13199-bib-0025] Watson, R. O. , & Galan, J. E. (2005). Signal transduction in *Campylobacter jejuni* induced cytokine production. Cellular Microbiology, 7(5), 655–665. 15839895 10.1111/j.1462-5822.2004.00498.x

[cmi13199-bib-0026] Wösten, M. M. , Boeve, M. , Koot, M. G. , van Nuenen, A. C. , & van der Zeijst, B. A. (1998). Identification of *Campylobacter jejuni* promoter sequences. Journal of Bacteriology, 180(3), 594–599. 9457862 10.1128/jb.180.3.594-599.1998 PMC106926

[cmi13199-bib-0027] Wren, B. W. , Stabler, R. A. , Das, S. S. , Butcher, P. D. , Mangan, J. A. , Clarke, J. D. , … Stoker, N. G. (1998). Characterization of a haemolysin from *Mycobacterium tuberculosis* with homology to a virulence factor of *Serpulina hyodysenteriae* . Microbiology, 144(Pt5), 1205–1211. 9611795 10.1099/00221287-144-5-1205

[cmi13199-bib-0028] Yusupov, M. M. , Yusupova, G. Z. , Baucom, A. , Lieberman, K. , Earnest, T. N. , Cate, J. H. , & Noller, H. F. (2001). Crystal structure of the ribosome at 5.5 Å resolution. Science, 292(5518), 883–896. 11283358 10.1126/science.1060089

[cmi13199-bib-0029] Zhang, Z. W. , Dorrell, N. , Wren, B. W. , & Farthingt, M. J. (2002). *Helicobacter pylori* adherence to gastric epithelial cells: A role for non‐adhesin virulence genes. Journal of Medical Microbiology, 51(6), 495–502. 12018657 10.1099/0022-1317-51-6-495

[cmi13199-bib-0030] Zheng, J. , Meng, J. , Zhao, S. , Singh, R. , & Song, W. (2008). *Campylobacter*‐induced interleukin‐8 secretion in polarized human intestinal epithelial cells requires *Campylobacter*‐secreted cytolethal distending toxin‐ and Toll‐like receptor‐mediated activation of NF‐kappaB. Infection and Immunity, 76(10), 4498–4508. 18644884 10.1128/IAI.01317-07 PMC2546826

